# Glycophenotyping of osteoarthritic cartilage and chondrocytes by RT-qPCR, mass spectrometry, histochemistry with plant/human lectins and lectin localization with a glycoprotein

**DOI:** 10.1186/ar4330

**Published:** 2013-10-04

**Authors:** Stefan Toegel, Daniela Bieder, Sabine André, Friedrich Altmann, Sonja M Walzer, Herbert Kaltner, Jochen G Hofstaetter, Reinhard Windhager, Hans-Joachim Gabius

**Affiliations:** 1 Karl Chiari Lab for Orthopaedic Biology, Department of Orthopaedics, Medical University of Vienna, Waehringer 18-20 1090 Vienna, Austria; 2 Institute of Physiological Chemistry, Faculty of Veterinary Medicine, Ludwig-Maximilians-University Munich, Munich, Germany; 3 Department of Chemistry, University of Natural Resources and Life Sciences, Vienna, Austria; 4 2nd Department, Orthopaedic Hospital Vienna-Speising, Vienna, Austria

## Abstract

**Introduction:**

This study aimed to characterize the glycophenotype of osteoarthritic cartilage and human chondrocytes.

**Methods:**

Articular knee cartilage was obtained from nine osteoarthritis (OA) patients. mRNA levels for 27 glycosyltransferases were analyzed in OA chondrocytes using RT-qPCR. Additionally, N- and O-glycans were quantified using mass-spectrometry. Histologically, two cartilage areas with Mankin scores (MS) either ≤4 or ≥9 were selected from each patient representing areas of mild and severe OA, respectively. Tissue sections were stained with (1) a selected panel of plant lectins for probing into the OA glycophenotype, (2) the human lectins galectins-1 and -3, and (3) the glycoprotein asialofetuin (ASF) for visualizing β-galactoside-specific endogenous lectins.

**Results:**

We found that OA chondrocytes expressed oligomannosidic structures as well as non-, mono- and disialylated complex-type N-glycans, and core 2 O-glycans. Reflecting B4GALNT3 mRNA presence in OA chondrocytes, LacdiNAc-terminated structures were detected. Staining profiles for plant and human lectins were dependent on the grade of cartilage degeneration, and ASF-positive cells were observed in significantly higher rates in areas of severe degeneration.

**Conclusions:**

In summary, distinct aspects of the glycome in OA cartilage are altered with progressing degeneration. In particular, the alterations measured by galectin-3 and the pan-galectin sensor ASF encourage detailed studies of galectin functionality in OA.

## Introduction

The emerging concept of the ‘sugar code’ has fundamentally changed our understanding of the significance of glycosylation [1]. Structures that were initially seen as an appendix solely modulating physicochemical properties of proteins turned out to be bioactive with high-density coding capacity. In fact, glycans of cellular glycoconjugates are intimately involved in diverse processes of cell-cell and cell-matrix interactions [1]. Work on hereditary diseases and murine models with engineered genetic deficiencies in glycosylation has revealed ample connections to apparent dysfunctions [2,3]. Insights into T cell activation and tumor suppressor-dependent changes of glycogene (glycosyltransferases, lectins) expression, for instance, have exemplified how swift reprogramming of distinct aspects of the glycophenotype elicits growth regulation [4-11]. Among others, these cases demonstrate that substitutions of the N-glycan core, known to act as switches for *cis*/*trans*-interactions [1,12], and the status/linkage type of sialylation are particularly prone to marked regulation: a result that guides the selection of tools (for example, lectins [13]) to monitor these aspects of the glycophenotype under disease conditions.

Osteoarthritis (OA), clinically characterized by pain, stiffness, joint effusion and loss of joint function/mobility, is a degenerative joint disease whose onset can depend on genetic, constitutional and biomechanical risk factors. Major cellular hallmarks of OA pathobiology include hypertrophic differentiation or apoptosis of chondrocytes, impaired cell adhesion and pro-inflammatory signaling that promotes the breakdown of the cartilage extracellular matrix [14]. Little is known, however, about the role of the cellular glycophenotype in the onset and progression of OA. In previous reports, we have focused on the characterization of glycan expression of immortalized human chondrocytes and cells from primary cultures [15,16]. Complemented with transcriptional profiling of selected glycosyltransferases and identification of the most abundant N/O-glycans, we described the impact of proinflammatory cytokines interleukin-1β and tumor necrosis factor-α on the glycan profile of chondrocytes [17,18]. The *in vitro* nature of these studies, however, did not allow addressing the role of modulated glycosylation in joint disease. The present work was therefore designed to test the hypothesis that the glycosylation signature of chondrocytes and extracellular matrix is affected in OA cartilage *in vivo* during disease progression. Accordingly, we here report on the lectin histochemical analysis of sections from articular cartilage of OA patients. In addition to the plant lectins listed in Table 1, we tested human lectins as probes, with the intention of defining chondrocyte reactivity for galectins, known to be endogenous adhesion/growth-regulatory effectors on the cell surface and intracellularly [19]. When labeled, these probes enable the delineation of the status and any alterations of cellular binding capacity, with the potential to detect disease-associated changes [5,20,21]. Homodimeric proto-type galectin-1 (Gal-1) and chimera-type galectin-3 (Gal-3) were selected, because Gal-1 is implicated in the regulation of chondrocyte growth/catabolism, while Gal-3 appears to exert a protective role on articular cells [22-24]. Of note, Gal-1 is an abundant cellular protein in human mesenchymal stem cells, supposedly relevant for cell-matrix interactions already at this early stage of development [25]. Its avian orthologue CG-1A participates, at a very early stage, in the formation and patterning of precartilage mesenchymal condensations in the developing limb, indicating fundamental functionality across phylogenetic boundaries [26]. Gal-3 has received attention in histopathology due to its diagnostic potential for thyroid lesions and, of note, can competitively interfere with Gal-1 activities [27-29]. Finally, we introduce asialofetuin (ASF), a glycoprotein that presents β-galactosides for lectin binding [30], to localize respective tissue lectins in cartilage *in situ.* The application of carrier-immobilized carbohydrate ligands in lung tumor sections had disclosed a correlation of binding to prognosis, intimating biological relevance beyond mapping [31]. Using plant and endogenous lectins as well as the labeled glycoproteins, our results define the glycophenotype in OA cartilage focusing on function-oriented aspects, and illustrate the feasibility of visualizing carbohydrate-binding capacity in this system glycohistochemically.

**Table 1:** Panel of lectins (plant agglutinins and human galectins) used for glycophenotyping of human OA cartilage

**Plant and lectins**	**Abbreviation**	**Monosaccharide specificity**	**Optimized concentration (ng/μl)**	**Potent oligosaccharide/glycoprotein glycan ligands**
*Canavalia ensiformis (jack bean) agglutinin*	ConA	Man/Glc	0.5	Manα6(Manα3)Manβ4GlcNAcβ4GlcNAc
*Pisum sativum* (pea) agglutinin	PSA	Man/Glc	0.5	N-glycan binding enhanced by core fucosylation
*Phaseolus vulgaris* (kidney bean) erythroagglutinin	PHA-E	^**a**^	1	Bisected complex-type N-glycans: Galβ3/4GlcNAcβ2Manα6(GlcNAcβ2-Manα3)(GlcNAcβ4)Manβ4GlcNAcβ4GlcNAc
*Phaseolus vulgaris* (kidney bean) leukoagglutinin	PHA-L	^**a**^	1	Tetra- and triantennary N-glycans with β6-branching
*Viscum album* (mistletoe) agglutinin	VAA	Gal	0.5	Galβ3(4)GlcNAc without/with α2,6-sialylation, Galα3(4)Gal, Galβ2(3)Gal, Fucα2Gal
*Lycopersicon esculentum* (tomato) agglutinin	LEA	^**a**^	2	core and stem regions of high-mannose-type N-glycans, (GlcNAcβ3Galβ4GlcNAcβ3Gal) repeats (LacDiNAc to polyLacNAc)
*Maackia amurensis* agglutinin-I (leukoagglutinin)	MAA-I	^**a**^	20	Neu5Ac/Gcα3Galβ4GlcNAc/Glc^**b**^, 3′-sulfation instead of sialylation and 9′-*O*-acetylation tolerated
*Sambucus nigra* (elderberry) agglutinin	SNA	Gal/GalNAc	0.4	Neu5Ac/Gcα6Gal/GalNAc^**c**^, clustered T_n_-antigen, 9′-*O*-acetylation tolerated
*Dolichos biflorus* (horse gram) agglutinin	DBA	GalNAc	50	GalNAcα3GalNAcα3Galβ4Galβ4Glc, clustered T_n_-antigen, histo-blood group A- tetrasaccharide, β-linked GalNAc in Sd^a^ antigen
*Arachis hypogaea* (peanut) agglutinin	PNA	Gal	0.7	Galβ3GalNAcα/β
*Artocarpus integrifolia* (jack fruit) agglutinin	Jacalin (JAC)	Gal/GalNAc	0.7	Galβ3GalNAcα, sialylation of T/T_n_ antigens tolerated
Galectin-1	Gal-1	^**d**^	1	Type I/II disaccharides (α2,3-sialylation/sulfation tolerated), LacNAc repeats (terminal α2,6-sialylation not tolerated), Fucα2Gal, extended core 2/4 structures, multiantennary N-glycans
Galectin-3	Gal-3	^**d**^	1	Type I/II and core 1 disaccharides (α2,3-sialylation/sulfation tolerated), LacNAc repeats (terminal α2,6-sialylation tolerated), GalNAcβ4GlcNAc (LacdiNAc), Galα3Galβ4GlcNAc, histo-blood group ABH epitopes, multiantennary N-glycans/clustered T-antigen

^**a**^no monosaccharide known as ligand; ^**b**^binding specific for type II LacNAc (Galβ4GlcNAc) core [32]; ^**c**^binding of type I LacNAc (Galβ3GlcNAc) core preferred, 6′-sulfation of GlcNAc in α2,6-sialylated LacNAc (type I/II) enhances affinity [33]; ^**d**^extension of Gal core to disaccharide (mostly in β-linkage such as Galβ2Gal or Galβ4Glc) required.

## Methods

### Clinical specimens

Human articular cartilage was obtained during total knee replacement surgeries in patients with OA (n = 9) with informed consent and in accordance with the terms of the ethics committee of the Medical University of Vienna (EK-No.: 1065/2011). Details on clinical specimens are given in Additional file 1: Table S1.

### Cell culture

Primary human OA chondrocytes were enzymatically isolated from femoral condyles and tibial plateaus of articular OA cartilage (n = 5) following established protocols [16-18]. Isolated chondrocytes were cultured in (Dulbecco’s) modified Eagle’s medium ((D)MEM; Gibco, Lofer, Austria) containing 10% fetal calf serum (FCS; Biochrom, Berlin, Germany) and 2 μl/ml gentamycin (Biochrom) in a humidified atmosphere of 5% CO_2_/95% air at 37°C. For all assays, only freshly isolated and seeded cells without subculturing were used.

### Quantitative real-time RT-PCR

Chondrocytes were grown in 12-well tissue culture plates (Iwaki, Tokyo, Japan) to 90% confluence. Total RNA was extracted using the NucleoSpin RNA II Kit (Macherey-Nagel, Düren, Germany). Each sample was run on the Agilent 2100 Bioanalyzer Nano LabChip for quality control and quantification of total RNA prior to reverse transcription into cDNA using the high capacity cDNA reverse transcription kit (Applied Biosystems, Vienna, Austria). RNA integrity numbers were between 9.6 and 10.

SYBR-green based qPCR assays for the glycosyltransferase transcripts were used as described previously [17,18]. The primers for B4GALNT3 (NM_173593; Forward: TGTTGAGATGGCACTGAAGAG; Reverse: TGGAGGTCACAGAGGAAGATG), an enzyme responsible for producing GalNAcβ4GlcNAc (LacdiNAc) termini [34], were designed using AlleleID software. In melting curve analysis, only one peak was observed confirming target specificity. Amplification efficiencies of primers were assessed using dilution series of cDNA prepared from chondrocyte mRNA. mRNA expression levels were calculated as relative copy numbers considering actual amplification efficiencies and with respect to that of glyceraldehyde-3-phosphate dehydrogenase (GAPDH) set at 1,000. Technically, the protocol deliberately followed the minimal guidelines for the design and documentation of qPCR experiments as recently outlined [35]. A qPCR checklist listing all relevant information is provided to assess the technical adequacy of the used qPCR protocols (see Additional file 2: Table S2).

### Protein and glycan preparation, quantification of oligosaccharides using LC-ESI-MS

The quantification of oligosaccharides using liquid chromatography-electrospray ionization-mass spectrometry (LC-ESI-MS) essentially followed previously described protocols [17,18]. Briefly, cultured OA chondrocytes were lysed and precipitated proteins were subjected to SDS-PAGE. Free N-glycans were obtained after trypsin and PNGase F digestions. O-Glycans were released by reducing β-elimination and further analyzed as done for borohydride-reduced N-glycans. Analysis of the glycans (from an aliquot equivalent to 2.5 × 10^5^ cells) by positive-ion LC-ESI-MS was performed with a 100 × 0.32 mm porous graphite carbon (PGC) column (Thermo, Vienna, Austria) at a flow rate of 5 μl/min maintained with a Dionex Ultimate 3000 cap flow system. Mass spectrometry was done using a Waters Q-TOF Ultima Global mass spectrometer with standard ESI source and MassLynx V4.0 SP4 software. The peak heights of the deconvoluted spectra as generated with the MaxEnt3 routine of MassLynx V4.0 served as measures for relative molar abundance.

### Histological assessment

For immunohistochemistry, tissue specimens from femoral condyles were selected macroscopically to provide one area of mild and one area of severe degeneration whenever possible. The specimens were fixed in formalin and decalcified using Titriplex-Tris-Solution (Gatt-Koller, Absam, Austria) prior to embedding in paraffin according to standard procedures. Paraffin sections (2.5 μm) were stained with safranin-O (Sigma, Vienna, Austria) and counter-stained using light-green Goldner III solution (Morphisto, Frankfurt, Germany). Using the Mankin scale, the degree of cartilage degeneration in the sections was graded according to histological and histochemical characteristics [36]. The Mankin score (MS) considers abnormalities in cartilage structure, cell population, safranin O stain distribution and tidemark integrity, and results in a final grade ranging from 0 (most intact) to 14 (most degenerated). From each patient, two areas with MS ≤4 and MS ≥9 were selected, representing one area of mild and one area of severe degeneration, respectively. Consecutive sections were then processed for lectin histochemical staining as described below.

### Lectin histochemistry

All lectin probes used for glycan mapping and their respective carbohydrate specificities are listed in Table 1. The plant lectins DBA, JAC, LEA, MAA-I, PHA-E, PHA-L, PNA and SNA were obtained as biotinylated probes from Vector Labs Burlingame, CA, USA. The *Viscum album* agglutinin (VAA; from extracts of leaves [37]) and the galectins (from recombinant production) were purified by affinity chromatography on lactosylated Sepharose 4B, obtained after divinyl sulfone activation, ConA and PSA (from seeds) on mannosylated Sepharose 4B, biotinylated under activity-preserving conditions and rigorously checked for maintained activity by solid-phase/cell assays as described [5,20,38]. Bovine fetuin was chemically desialylated by acid/heat treatment to yield ASF, which was biotinylated as described [39], followed by ascertaining galectin reactivity in solid-phase assays using labeled Gal-1/-3 as sensors.

Performing titration experiments, optimal lectin concentrations that yielded best signal-to-background ratios were determined and kept constant for processing within this comparative study (all listed in Table 1). Following deparaffinization, the tissue sections were washed twice with PBS for 5 minutes, then exposed to PBS/H_2_O_2_ (200 ml PBS mixed with 6 ml 30% H_2_O_2_) to block endogenous peroxidase activity for three minutes and finally washed again twice with PBS. The biotinylated lectins were diluted with 2% BSA/HEPES and the biotinylated galectins were diluted with 2% BSA/PBS. Epitope-independent binding was blocked by preincubation with the respective BSA solutions for 30 minutes at room temperature. The sections were then incubated overnight with the biotinylated probes at 4°C and, after thorough washing, developed using the VECTASTAIN Elite ABC Kit (Vector Labs) with NovaRED peroxidase substrate kit (Vector Labs). Counterstaining was performed using Mayer’s hemalum solution (Merck, Vienna, Austria). Thereafter, sections were thoroughly rinsed and mounted for microscopy. The omission of biotinylated probes from the process or competitive inhibition (by lactose) was performed to test the carbohydrate dependence of lectin histochemistry. The samples were evaluated using an Olympus Vanox AHBT3 microscope and the images were processed using cell^D software (Olympus). Staining intensities above background levels were scored as positive. The staining of chondrons and interterritoreal matrix of mildly degenerated (MS ≤4; n = 7) and severely degenerated (MS ≥9; n = 9) areas was assessed independently by two observers. Cooperative analysis of discordant slides led to consensus in all cases.

The percentage of ASF-reactive chondrocytes in the areas of mild and severe degeneration was determined by evaluating the fraction of stained cells at a magnification of 20× on the basis of 100 chondrocytes per area (starting from the cartilage surface). For statistical analysis of the data, a paired *t*-test following control for Gaussian distribution was performed using the Microsoft Excel integrated analysis tool. In accordance with recent guidelines [40], all analysis units (n) given in figure and table legends refer to the number of independent observations (biological replicates) underlying the respective descriptive statistics and statistical tests.

### Glycocytochemistry

OA chondrocytes were seeded on glass coverslips placed into 24-well plates. Cells were fixed with 400 μl ice-cold methanol for 20 minutes and rehydrated in 700 μl PBS for 20 minutes at room temperature. Cells were incubated with 250 μl of solution containing biotinylated ASF (20 μg/ml in 1% BSA/PBS) for 1 hour at 37°C. After three washing steps with PBS, cells were incubated for 1 hour at 37°C with streptavidin-PE (diluted 1:40 in 1% BSA/PBS; Sigma) and 300 nM DAPI (Invitrogen, Darmstadt, Germany). After washing three times with PBS, cells were embedded in FluorSaveTM Reagent (Calbiochem, San Diego, CA, USA) for microscopic inspection at 62× magnification using a Carl Zeiss LSM 700 Laser Scanning Microscope and Zen software.

## Results

### Expression of selected glycosyltransferase genes

As a first parameter of the glycophenotype of OA chondrocytes, mRNA levels of glycosyltransferases were quantified using RT-qPCR (for details on enzyme functions, please see the legend of Table 2; for further information, please see Carbohydrate-Active Enzyme (CAZy) database [41]). As listed in Table 2, we included key enzymes involved in processing, branching and sialylation of N-glycans and O-glycans, along with three chondrocyte markers as internal controls as previously reported [16]. The rich levels of COL2 and ACG as well as the differentiation index COL2/COL1 ascertained the chondrocyte phenotype of cultured cells in this study.

**Table 2:** Presence of mRNA for selected glycosyltransferases in OA chondrocytes

		** N-glycans**			
*MAN1C1*	0.8	*MGAT4A*	172.6	*ST6Gal1*	676.8
*MAN2A1*	449.9	*MGAT4B*	77.0	*ST6Gal2*	5.2
*MGAT1*	723.3	*MGAT5A*	387.3	*ST3Gal3*	59.4
*MGAT2*	2,328.2	*MGAT5B*	2.9	*ST3Gal4*	153.7
*MGAT3*	10.1	*FUT8*	263.9	*ST3Gal6*	14.7
		*B4GALNT3*	104.4		
	** O-glycans**			** Chondrocyte markers**	
*GALNT*	3,925.3	*ST3Gal1*	60.4	*AGC*	1,704.7
*B3GNT*	141.8	*ST3Gal2*	75.7	*COL2*	9,953.7
*GCNT1*	375.5	*ST6GalNAc1*	n.d.	*COL1*	5,722.9
*FUT1*	0.4	*ST6GalNAc2*	n.d.		
*3OST*	19.4	*ST6GalNAc3*	0.7		
		*ST6GalNAc4*	9.8		

Distinct mRNA species were quantified using RT-qPCR. Numbers denote relative copy numbers with respect to the expression of the GAPDH gene arbitrarily set at 1,000. cDNA of OA chondrocytes from five donors was pooled and analyzed in duplicate. All series of measurements (technical replicates) had a standard deviation below 1.5%.

MAN2A1 and MGAT2: conversion of oligomannosides to complex-type N-glycans. MGAT1: committing step for synthesis of hybrid- and complex-type N-glycans. MAN1C1: trimming of oligomannosidic structures. MGAT3: introduction of bisecting GlcNAc to the core of complex-type glycans in β1,4-linkage. MGAT4 and MGAT5: production of tri- and tetra-antennary N-linked sugar chains. FUT8: transfer of fucose to the core of complex-type glycans in α1,6-linkage. B4GALNT3: β1,4-N-acetylgalactosaminyltransferase 3, forming the LacdiNAc terminus. ST6GAL1, ST6GAL2: N-glycan α2,6-sialyltransferases. ST3GAL3, ST3GAL4, ST3GAL6: N-glycan α2,3-sialyltransferases. GALNT1: initiates O-linked mucin-type glycosylation in the Golgi apparatus. B3GNT2: a major poly-N-acetyllactosamine synthase. GCNT: formation of the core 2 O-glycan branch. FUT1: O-glycan fucosyltransferase. 3OST: sulfotransferase for the 3′-position of galactose. ST3GAL1 and ST3GAL2: O-glycan α2,3-sialyltransferases. ST6GALNAC1, ST6GALNAC2, ST6GALNAC3 and ST6GALNAC4: O-glycan α2,6-sialyltransferases acting on GalNAc as acceptor. AGC: aggrecan. COL2: collagen type-II. COL1: collagen type-I. n.d.: not detectable.

The enzyme profile let us expect synthesis of complex-type N-glycan (MAN2A1, MGAT2) with core substitutions (FUT8, MGAT3) and branching (MGAT4, MGAT5). The considerable level of B4GALNT3 expression by OA chondrocytes suggested the presence of LacdiNAc termini in N-glycans of OA chondrocytes. Of note for O-glycans, expression of GALNT1 (from the complexity of this enzyme family, we focused on this representative major member [42,43]), B3GNT2 and GCNT enables production of core 2 glycans with LacNAc repeats. Sialylation can also be expected (Table 2).

In principle, these data reveal fulfillment of an essential prerequisite for glycan biosynthesis but should not be interpreted to reliably predict quantitative aspects of actual glycosylation. Correct positioning of the gene products within the glycosylation machinery and availability of substrates, among others, are factors playing into the production of glycans. Thus, to provide insights into the glycome, we monitored the presence of glycans by LC-ESI-MS.

### Profiling of N-and O-glycans

**Figure 1:**
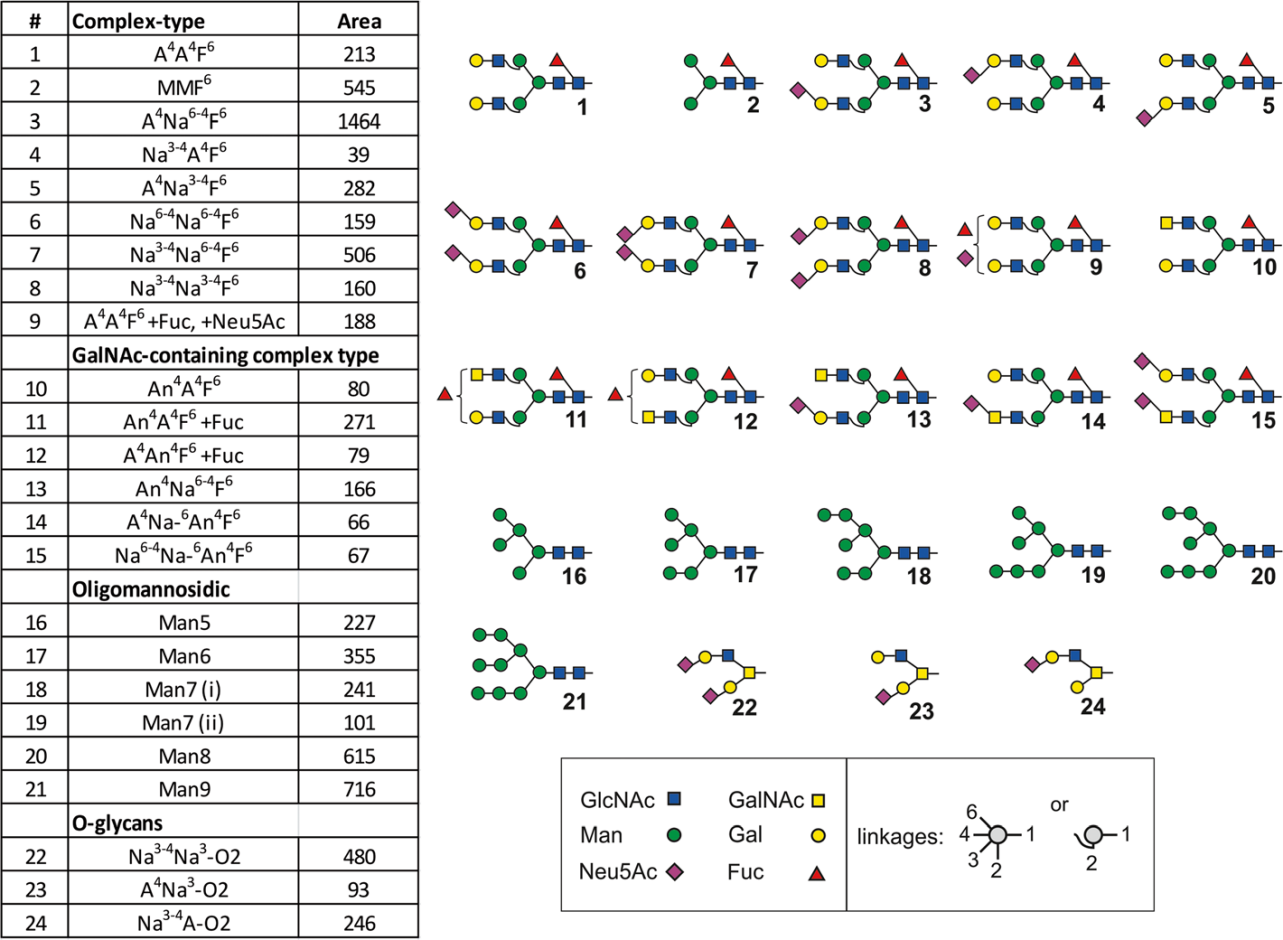
**Major glycan structures found in OA chondrocytes.** Major N-glycan and O-glycan species, that is, oligomannosidic structures, non-, mono- and disialylated N-glycans, β4GalNAc-containing N-glycans as well as mucin-type core 2 O-glycans, were identified and quantified independently in chondrocytes from three patients by LC–ESI-MS. Results shown were obtained from chondrocytes of one patient (n = 1) and are representative of three independent experiments with similar results. Glycans are referred to according to the ‘proglycan’ nomenclature (http://www.proglycan.com). The oligosaccharides shown here were selected for quantification with LC-ESI-MS, because they are among the most abundant glycan structures in human chondrocytes and are essentially absent in the glycome of the fetal calf serum-containing cell culture medium, which can otherwise contaminate the chondrocyte glycome to some extent. As a measure of quantity the mean peak area values obtained by LC–ESI-MS are given for each structure. For each peak area, the standard deviation resulting from two technical replicates was below 15%. LC–ESI-MS, liquid chromatography-electrospray ionization-mass spectrometry; OA, osteoarthritis.

Structures of the glycoprotein-derived N- and O-glycans from OA chondrocytes are listed in Figure 1, together with quantitative data, with emphasis on glycans not present in the culture medium to avoid contamination. Meeting the expectations from RT-qPCR profiling, the glycan population detected includes core-substituted, α2,3/6-sialylated N-glycans, with the presence of LacdiNAc-terminated structures and core 2 O-glycans. This mapping provides a quantitative view of glycan structures presented by OA chondrocytes. Together with the data in Table 2, these results allowed us to strategically set up the panel of plant lectins for probing into the OA glycophenotype, in terms of presence/absence of distinct determinants and spatial aspects (for a survey of lectin specificities, please see Table 1).

### Glycophenotyping by plant lectins

**Figure 2:**
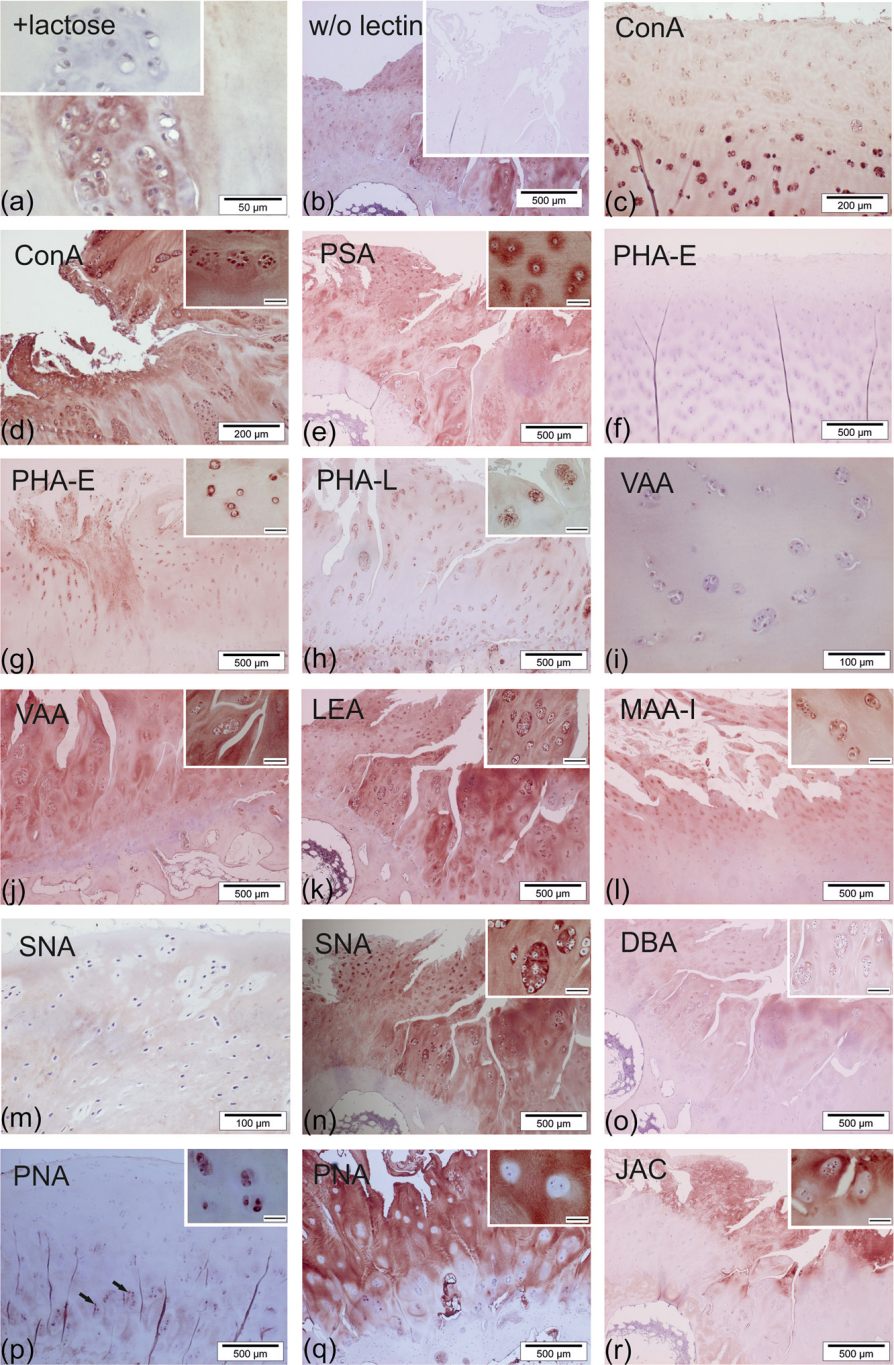
**Lectin histochemical staining profiles in sections of OA cartilage. (a)** Binding of PNA to complex chondrons of a severely degenerated cartilage region could be completely blocked with lactose (inset) ascertaining carbohydrate-specific binding. **(b)** Omission of the incubation step with biotinylated LEA (first-step reagent) from processing excluded probe-independent signal generation. **(c, d)** ConA staining: staining pattern of MS ≤4 regions included chondrocytes in deep zones of cartilage **(c)**. Intense staining of matrix and chondrons (inset) in MS ≥9 regions **(d)**. **(e)** PSA staining: positivity of chondrons (inset) and matrix, predominantly in MS ≥9 cartilage. **(f, g)** PHA-E staining: whereas MS ≤4 regions were negative **(f)**, MS ≥9 areas **(g)** presented positive chondrons (inset) and matrix. **(h)** PHA-L staining: binding sites were restricted to chondrons (insert) and matrix of MS ≥9 cartilage. **(i-j)** VAA staining: whereas the chondrons of MS ≤4 areas were negative **(i)**, reactivity was observed both in chondrons (inset) and matrix of MS ≥9 cartilage **(j)**. **(k)** LEA staining: reactivity for chondrons (inset) and matrix of MS ≥9 regions. **(l)** MAA-I staining: reactivity included chondrons (inset) and matrix of MS ≥9 cartilage. **(m, n)** SNA staining: weak staining of matrix and no staining of chondrons in MS ≤4 cartilage **(m)**, whereas both chondrons (inset) and matrix were positive in MS ≥9 regions **(n)**. **(o)** DBA staining: positivity in chondrons (inset) and matrix of MS ≥9 cartilage. **(p, q)** PNA staining: positive chondrocytes sparely distributed in the deeper zones of MS ≤4 cartilage (**p**; arrows, inset). In MS ≥9 cartilage **(q)**, intense matrix staining was observed, whereas chondrons were mostly negative (inset). **(r)** JAC staining: absent in chondrons (inset), but present in superficial zones of MS ≥9 cartilage. Bars in inserts of d,e,g,l,n,p,q,r: 50μm. Bars in inserts of **h**,**j**,**k**,**o**: 100μm. MS, Mankin score; OA, osteoarthritis.

Each lectin was systematically tested by titration to determine the optimal concentration for reaching the best signal-to-background ratio. At this concentration, which is listed in Table 1, inhibition by cognate sugar ascertained the reactivity, as illustrated in Figure 2a for blocking typical binding to chondrocytes and extracellular matrix. In addition, omission of the incubation step with labeled lectin from routine processing enabled us to exclude lectin-independent signal generation (Figure 2b). Additional file 3: Table S3 presents a quantitative overview of lectin-binding patterns, as a function of the MS. In detail, flanked by respective representative illustrations, lectin binding was characterized as follows:

Con A, in contrast to the other lectins, stained all mildly and severely degenerated cartilage regions. Chondrons in both mildly (100%; 7/7) and severely degenerated (100%; 9/9) specimens were positive (see Additional file 3: Table S3). Cells were stained in the middle and deeper zones in mildly degenerated areas (Figure 2c), whereas severely degenerated areas showed cell staining throughout the entire cartilage (Figure 2d), strong reactivity of blood vessels and osteocytes was observed, and the subchondral bone matrix remained unstained (not shown).

PSA, reactive with core-fucosylated N-glycans, stained OA cartilage with a quantitative difference according to the MS (see Additional file 3: Table S3). In the majority of severely degenerated areas, the interterritorial matrix presented intense reactivity (Figure 2e). Comparable to ConA, the subchondral bone matrix was negative, whereas osteocytes and blood vessels bound the lectin (not shown).

PHA-E stained chondrocytes in mildly degenerated regions (2/7; 29%) less frequently and intensely than in severely degenerated regions (5/9; 56 %; Figure 2f, g; Additional file 3: Table S3). The interterritorial matrix was negative in less degenerated cartilage (0/7; 0%). but reactive in more degenerated areas (4/9; 44%). Pannus-like tissue, whenever present, as well as osteocytes and blood vessels showed reactivity for PHA-E, whereas subchondral bone matrix was not stained (not shown).

PHA-L neither bound to chondrocytes nor to interterritorial matrix of mildly degenerated cartilage regions (0/7; 0%). In comparison, positive chondrocytes and interterritorial matrix were detected in 22% (2/9) and 11 % (1/9) of severely degenerated areas, respectively (Figure 2h). Similar to PHA-E, PHA-L preferentially stained complex chondrons. Cells of the pannus-like tissue, blood vessels and parts of the subchondral bone matrix presented PHA-L reactivity (not shown).

VAA, negative in areas of mild degeneration (0/7; Figure 2i), reacted with chondrons and interterritorital matrix of severely affected regions (3/9; Figure 2j; Additional file 3: Table S3). The subchondral bone matrix and blood vessels were stained too, but not the pannus-like tissue (not shown).

LEA binding was selective for chondrons and interterritorial matrix in 22% (2/9) of severely degenerated cartilage regions (Figure 2k; Additional file 3: Table S3). In addition, the subchondral bone matrix was positive in the two reactive specimens. Less affected areas (0%; 0/7), pannus-like tissue, blood vessels and osteocytes were negative (not shown).

MAA-I did not react with chondrons and matrix of mildly degenerated cartilage areas (0/9; 0%). In 44% (4/9) of severely degenerated regions, reactivity for MAA-I was observed in the chondrons and, in 56% (5/9) of severely degenerated areas, the interterritorial matrix was also positive (Figure 2l; Additional file 3: Table S3). Pannus-like tissue was positive whenever present, whereas blood vessels or the subchondral bone presented no reactivity (not shown).

SNA was rarely reactive with chondrons and matrix in cartilage regions of mild degeneration (1/7; 14%; Additional file 3: Table S3, Figure 2m). In comparison, 44% (4/9) of severely degenerated areas presented reactive chondrons, while 22% (2/9) of these regions showed reactivity for interterritorial matrix (Figure 2n). Pannus-like tissue, blood vessels and subchondral bone were negative (not shown).

DBA stained chondrons only in areas of severe degeneration (22%; 2/9; Figure 2o; Additional file 3: Table S3). The interterritorial matrix presented staining in a comparable amount of areas of mild (29%; 2/7) and severe degeneration (33%; 3/9). Pannus-like tissue reactivity was observed in one out of two cases. Blood vessels and the subchondral bone were unstained throughout all samples (not shown).

PNA reacted with chondrons of all mildly degenerated cartilage regions (100%; 7/7; Figure 2p). Interestingly, only 67% (6/9) of the severely degenerated regions showed PNA-positive chondrons (see Additional file 3: Table S3). In contrast, interterritorial matrix was positive in only 43% (3/7) of less affected areas, whereas a marked increase and intensive staining was observed in more affected regions (100%; 9/9; Figure 2q). The pannus-like tissue gave signals whenever present. In five cases, PNA staining was observed in osteocytes, whereas in the subchondral bone matrix no staining was found (not shown).

JAC bound the interterritorial matrix in the proximity of surface fissures in three out of nine cases of severely degenerated regions (33%; Additional file 3: Table S3). Interestingly, chondrons were negative, resulting in characteristic, unstained regions around chondrocytes (Figure 2r). In those three specimens, the subchondral bone matrix was also positive. In contrast, mildly degenerated cartilage areas exhibited no staining of cells or matrix. Furthermore, pannus-like tissue, blood vessels or osteocytes were negative.

These data define the glycophenotype of OA cartilage with respect to distinct carbohydrate determinants. Moving from plant to human lectins as probes to strengthen the aspect of potential physiological implications, we next applied two human lectins, that is, Gal-1 and Gal-3, to the cartilage sections. In addition to their reactivity to glycans (please see Table 1 for details), these endogenous effectors can also react with distinct proteins intracellularly.

### Glycophenotyping with human galectins

**Figure 3:**
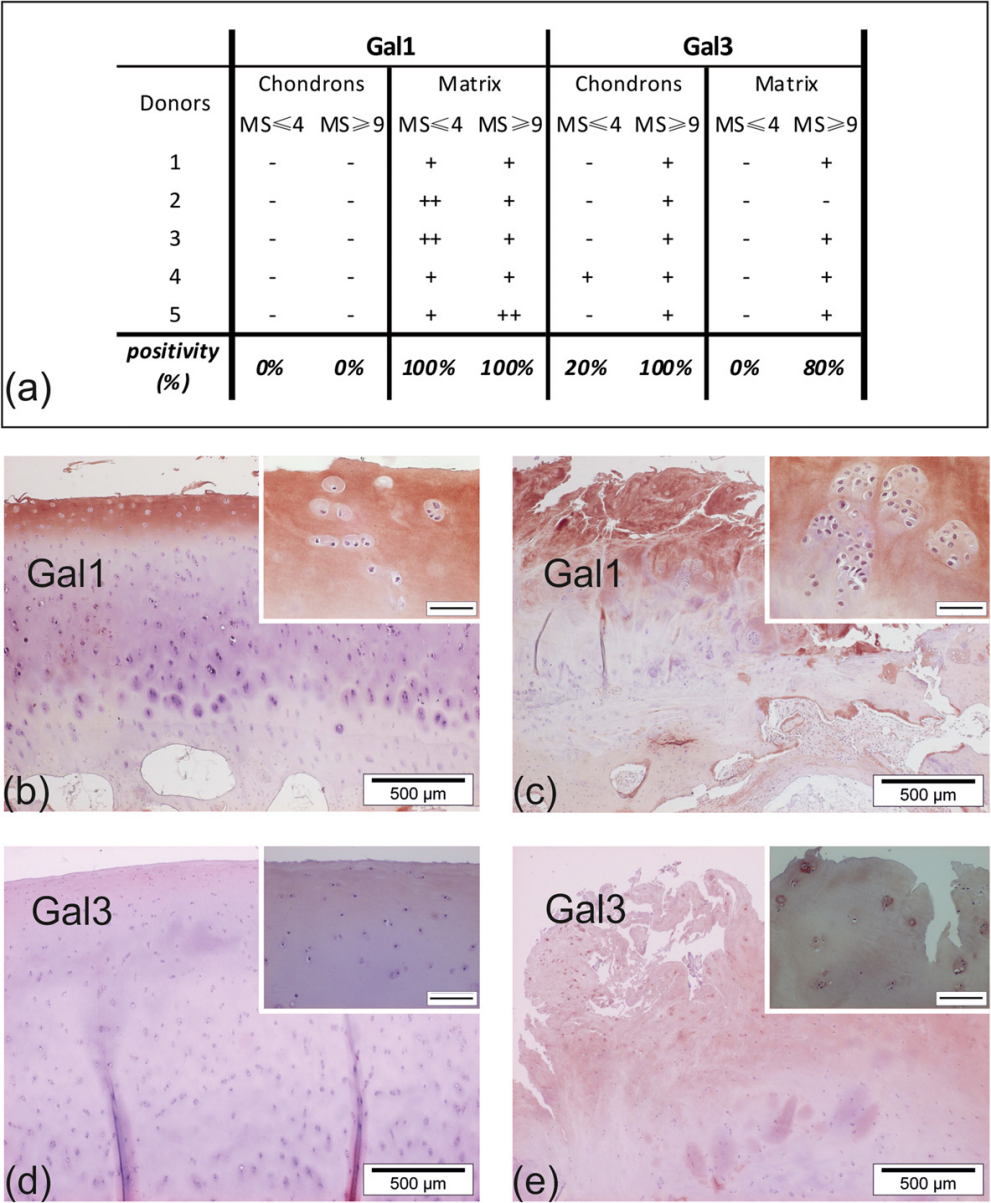
**Binding sites of human galectins in OA cartilage. (a)**: OA cartilage from five donors was histologically processed and stained with labeled Gal-1 and Gal-3, respectively. Positivity of chondrons and interterritorial matrix was assessed microscopically for MS ≥9 and MS ≤4 regions separately. The percentages given at the bottom of the table refer to the fractions of specimens presenting stained chondrons or interterritorial matrix among all analyzed OA cartilage specimens (n = 5). − no staining; + moderate staining; ++ intense staining; MS: Mankin score **(b, c)**: Gal-1 staining: reactivity included the matrix of MS ≤4 **(b)** and MS ≥9 **(c)** regions of OA cartilage. Insets show negativity of chondrons. **(d, e)**: Gal-3 staining: In MS ≤4 regions **(d)**, no staining of chondrocytes and matrix, whereas in MS ≥9 regions **(e)** reactivity for both chondrons and matrix was observed. Bars in inserts of **(b**-**e)**: 50μm. Gal-1, galectin-1, Gal-3, galectin-3, OA, osteoarthritis.

The interterritorial matrix of all mildly and severely degenerated cartilage areas was positive for Gal-1 (5/5, 100%; Figure 3a). In comparison to less affected areas (Figure 3b), however, most of the more degenerated areas exhibited an intense staining reactivity (Figure 3c). No staining was observed in chondrons, regardless of the degeneration status of cartilage. Moreover, osteocytes and the subchondral bone matrix were negative, whereas blood vessels were reactive (not shown).

Mildly degenerated cartilage, especially the matrix, was rarely positive for Gal-3 (Figure 3a, d). In contrast, this lectin stained – partly intensely – chondrons (5/5) and interterritorial matrix (4/5) of most severely degenerated cartilage specimens (Figure 3a, e). Pannus-like tissue, whenever present, was also positive.

Regarding the detection of LacdiNAc-presenting N-glycans and core 2 O-glycans it should be noted that LacdiNAc is known as ligand for Gal-3, for which core 2 branching is not a favorable factor [44,45]. Having herewith proven the presence of galectin-binding sites and illustrated their spatial distribution, we became interested to reveal whether tissue galectins were expressed in OA cartilage. Thus, we employed labeled ASF, known to serve as a pan-galectin sensor [30].

### Glycohistochemical analysis

**Figure 4:**
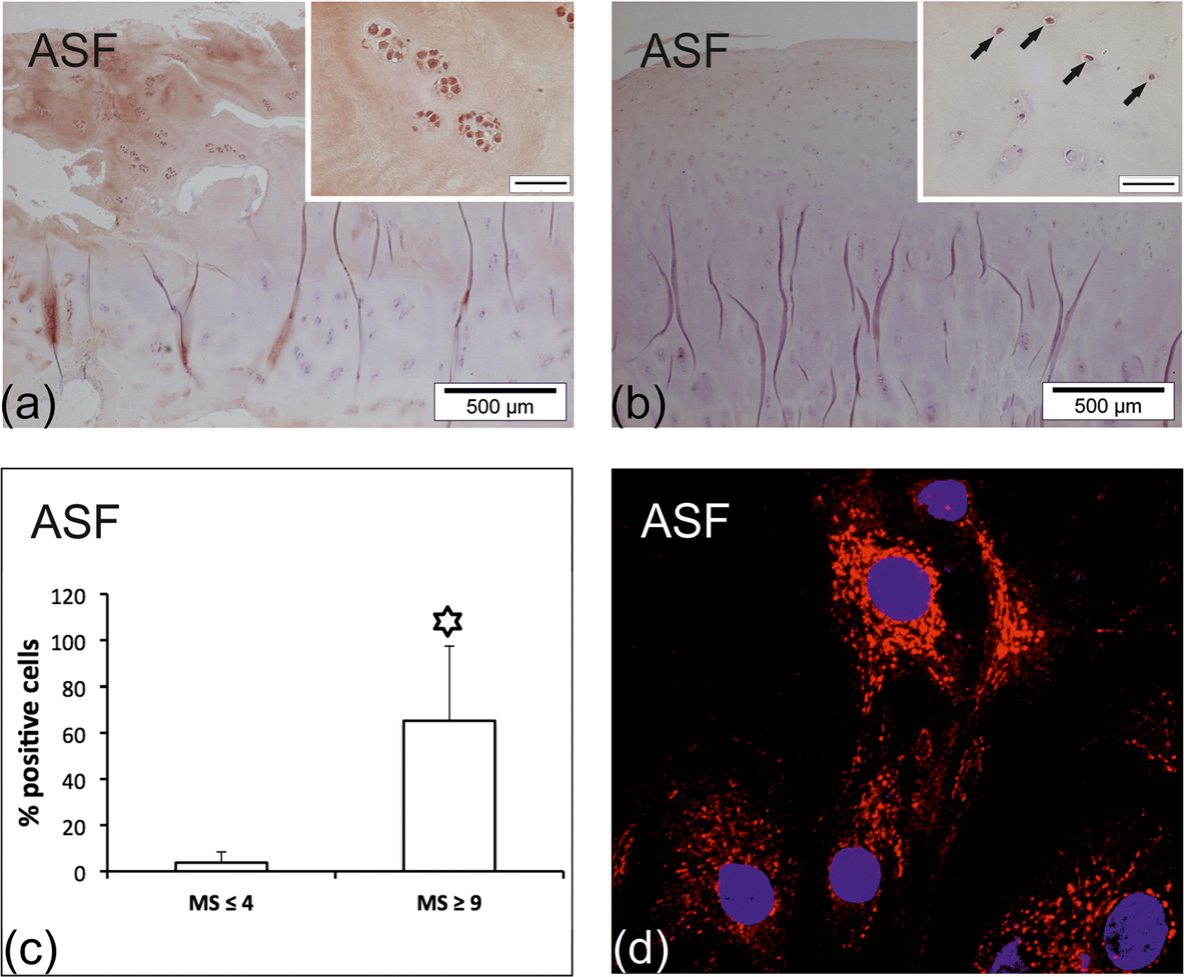
**Binding sites for ASF in human OA cartilage and chondrocytes. (a, b)** Reactivity for ASF in OA cartilage. **(a)** Intense reactivity of ASF with chondrocytes and matrix of MS ≥9 cartilage. The inset shows stained complex chondrons. **(b)** In MS ≤4 regions, positive chondrocytes were scattered across the superficial zone of cartilage (inset and arrows). Bars in insets of **(a)** and **(b)**: 50 μm. **(c)** Shown is a quantitative comparison between MS ≤4 and MS ≥9 regions of OA cartilage. Each bar represents the mean percentage of stained (reactive for ASF) chondrons in the respective areas (n = 7; see Additional file 3: Table S3). The asterisk indicates a significant difference of cell reactivity between cartilage areas of mild and severe degeneration (*P* = 0.0015; n = 7; paired *t*-test). **(d)** Subcellular distribution of ASF reactivity in cultured OA chondrocytes. Cells were incubated with biotinylated ASF and stained with streptavidin-phycoerythrin. ASF reactivity (red) was observed using laser scanning microscopy. Nuclei were counterstained with DAPI (blue). ASF, asialofetuin; DAPI, 4′,6-diamidino-2-phenylindole; MS, Mankin score; OA, osteoarthritis; PE, phycoerythrin.

Using sections of human quadriceps muscle as internal control for the reactivity of labeled ASF [46], application of this probe was optimized. Its enzymatic deglycosylation completely abolished binding in controls, underscoring the crucial role of glycan binding (not shown). The majority of arthritic cartilage areas (89%; 8/9) presented ASF staining of chondrons and interterritorial matrix (Figure 4a). In three cases, a particularly intense signal was observed, whereas one specimen did not respond to ASF (see Additional file 3: Table S3). In addition, reactivity was also observed in the pannus-like tissue. In areas of mild degeneration, chondrons and matrix were less frequently stained (43%; 3/7; Figure 4b). Subchondral bone was negative; blood vessels were weakly positive (not shown).

In quantitative comparison, ASF-positive cells were present in significantly higher rates in severely degenerated areas than in mildly degenerated areas (Figure 4c). At the subcellular level, ASF binding was seen in a fine-granular manner throughout the cytoplasm whereas the nucleus and the cell membrane were consistently negative (Figure 4d).

## Discussion

This study was designed to characterize the glycophenotype of OA cartilage using human chondrocytes *in vitro* and lectin histochemical analysis of clinical specimens. Initially, we monitored glycosyltransferase mRNA levels as well as N- and O-glycans (excluding glycans present in the used serum in the culture medium) by RT-qPCR and by LC-ESI-MS, respectively. The presented quantitative data, encouraging detailed glycogene monitoring (for example, β1,3/4-galactosyltransferases or glycohydrolases such as sialidases), gave reason for selecting a panel of plant lectins for the histochemical investigations. In this context, we focused on lectins targeting substitutions of the N-glycan core, which are potent switches for conformational behavior and reactivity for tissue lectins [12,47], presence of LacNAc repeats and the status of sialylation. Owing to previous indications for plant lectins (that is, ConA and wheat germ agglutinin) to act as probes for degenerative joint diseases [48], we also included ConA. The semiquantitative assessment of stained OA cartilage defined the presence and localization of plant-lectin-reactive glycan epitopes. An enzymatic pre-treatment to remove glycosaminoglycans was deliberately not performed to avoid non-physiological alteration of accessibility to probes, especially in view of the binding of the human galectins. Most importantly, it delineated differences with respect to the degeneration grade of cartilage within the patients (as determined using the MS) in certain cases. In particular, altered staining frequency and intensity were observed for lectins specific for (1) α2,3-sialylation of LacNAc-terminated N-glycans, (2) galactosides (reactive with VAA) and (3) bisected N-glycans (the bisecting GlcNAc residue acts on the local density of branch-end epitopes and may affect recognition processes directly or indirectly [47,49]). Since distinct sugar epitopes are the docking sites for tissue lectins to turn glycoconjugates into the ligand part of functional counter-receptors, we next applied two human adhesion/growth-regulatory lectins, that is, Gal-1 and Gal-3, as histochemical tools.

The respective results illustrate the feasibility of this application and show differences between the binding patterns of the two tested galectins. Whereas the interterritorial matrix of cartilage and blood vessels were the main sites of Gal-1 reactivity, chondrons and pannus-like tissue bound Gal-3. Of note, isolated OA chondrocytes, as previously observed for samples of rabbits with articular cartilage deterioration [50], presented N-glycans with the LacdiNAc terminus, a binding partner for Gal-3 but not for Gal-1.

Based on these findings, it can be postulated that galectins should be present in OA cartilage as on-site effectors that can translate the sugar code of cells and matrix into biological functions. The presence of binding sites for the glycoprotein ASF, whose glycans bind rather equally well to the human galectins [51], underscores this assumption. In fact, our data revealed an increase of ASF reactivity of OA cartilage as a function of the MS, suggesting elevated levels of galectins in areas of cartilage degeneration. Of relevance for joint diseases, Gal-3 was shown to localize in the synovium obtained from rheumatoid arthritis patients at the sites of cartilage and bone destruction, whereas Gal-1 was detected mostly in the sublining layer [52]. In addition, other galectins including galectins-8 and -9 appear of interest. Galectin-8 is widely expressed in human tissues and tumors [53,54] and, to emphasize potential orthopedic relevance, is produced and secreted by human synovial fluid cells in patients with rheumatoid arthritis [55]. Detection of autoantibodies against this and other galectins in sera of respective patients has been reported [56]. Interestingly, a clinical association of a single nucleotide polymorphism in the coding region of the galectin-8 gene (that is, the F19Y substitution) was recently revealed with rheumatoid arthritis [57].

## Conclusions

In summary, the present study adds to the characterization of the glycophenotype of chondrocytes and matrix in OA, with special emphasis on clinical specimens. In particular, it reports first insights into the reactivity of OA cartilage with tissue effectors and thereby gives further work on endogenous lectins a clear direction: systematic monitoring to define the localization of galectins by immunohistochemical fingerprinting in OA, along with monitoring binding-site availability and target glycoproteins, and the *in vitro* testing of galectins in relevant cell models.

## Abbreviations

ASF: Asialofetuin; BSA: Bovine serum albumin; ConA: *Canavalia ensiformis* agglutinin; DAPI: 4′,6-diamidino-2-phenylindole; DBA: *Dolichos biflorus* agglutinin; (D)MEM: (Dulbecco’s) modified Eagle’s medium; FCS: Fetal calf serum; Gal-1: Galectin-1; Gal-3: Galectin-3; GAPDH: Glyceraldehyde-3-phosphate dehydrogenase; HEPES: 4-(2-hydroxyethyl)-1-piperazineethanesulfonic acid; JAC: *Artocarpus integrifolia* agglutinin; LC-ESI-MS: Liquid chromatography-electrospray ionization-mass spectrometry; LEA: *Lycopersicon esculentum* agglutinin; MAA-I: *Maackia amurensis* agglutinin-I; MS: Mankin score; OA: Osteoarthritis; PBS: Phosphate buffered saline; PCR: Polymerase chain reaction; PHA-E: *Phaseolus vulgaris* erythroagglutinin; PHA-L: *Phaseolus vulgaris* Leucoagglutinin; PNA: *Arachis hypogaea* agglutinin; PSA: *Pisum sativum* agglutinin; RT-qPCR: Quantitative real-time PCR; SNA: *Sambucus nigra* agglutinin; VAA: *Viscum album* agglutinin.

## Competing interests

The authors declare that they have no competing interests.

## Authors’ contributions

ST and HJG conceived and designed the study and wrote the manuscript together with SA. SA and HK prepared the probes and performed respective quality controls. JGH and RW provided the clinical samples. ST, DB, SW and FA performed the experiments. ST, DB, SA, HK, FA and HJG analyzed and interpreted the data. All authors read and approved the final manuscript.

## Supplementary Material

Additional file 1: Table S1 Characteristics of clinical specimens. OA cartilage was obtained from OA patients (age range 54 to 80 years) between September 2010 and April 2012 according to the protocol given in the Methods section. Click here for file

Additional file 2: Table S2 MIQE checklist of RT-qPCR assays. Click here for file

Additional file 3: Table S3 Localization of lectin-reactive glycans in OA cartilage. Click here for file
