# Supplementary material for: Glycophenotyping of osteoarthritic cartilage and chondrocytes by RT-qPCR, mass spectrometry, histochemistry with plant/human lectins and lectin localization with a glycoprotein
Source: Arthritis Res Ther. 2013 Oct 4;15(5):R147. doi: 10.1186/ar4330 (PMC3978707; doi:10.1186/ar4330)
Supplement: Additional file 1: Table S1 — Characteristics of clinical specimens. OA cartilage was obtained from OA patients (age range 54 to 80 years) between September 2010 and April 2012 according to the protocol given in the Methods section. [file ar4330-S1.pdf]

#	Assay	Cause for surgery	Chronic Diseases
**1**	Histology	osteoarthritis of the left knee	arterial hypertension, COPD, nicotine abuse, osteoporosis, juvenile-onset diabetes, chronic prostatitis
**2**	Histology	osteoarthritis of the left knee	arterial hypertension, coronary heart disease, nicotine abuse, COPD, hyperlipoproteinemia, Raynaud‘s phenomenon, chronic renal insufficiency
**3**	Histology	osteoarthritis of the right knee	arterial hypertension, coronary heart disease, hyperlipidemia, adult onset diabetes
**4**	Histology	osteoarthritis of the left knee	arterial hypertension, obesity
**5**	Histology	osteoarthritis of the right knee	arterial hypertension, obesity, adult onset diabetes
**6**	Histology	osteoarthritis of the left knee	arterial hypertension
**7**	Histology	osteoarthritis of the left knee	obesity
**8**	Histology	osteoarthritis of the right knee	arterial hypertension, obesity, hypercholesteremia
**9**	Histology	osteoarthritis of the right knee	--
**10**	RT-qPCR; LC/MS	osteoarthritis of the left knee	arterial hypertension, obesity, adult onset diabetes, hypercholesteremia, hepatopathy
**11**	RT-qPCR; LC/MS	osteoarthritis of the right knee	--
**12**	RT-qPCR; LC/MS	osteoarthritis of the left knee	arterial hypertension
**13**	RT-qPCR	osteoarthritis of the right knee	--
**14**	RT-qPCR	osteoarthritis of the left knee	arterial hypertension, adult onset diabetes, hyperlipidemia
