# Supplementary material for: Glycophenotyping of osteoarthritic cartilage and chondrocytes by RT-qPCR, mass spectrometry, histochemistry with plant/human lectins and lectin localization with a glycoprotein
Source: Arthritis Res Ther. 2013 Oct 4;15(5):R147. doi: 10.1186/ar4330 (PMC3978707; doi:10.1186/ar4330)
Supplement: Additional file 3: Table S3 — Localization of lectin-reactive glycans in OA cartilage. [file ar4330-S3.pdf]

Table 3. Localization of lectin-reactive glycans in OA cartilage.

OA cartilage from nine donors was histologically processed and stained with labeled lectins and ASF. Specimens, except for specimens 6 and 7, encompassed areas of advanced (MS≥9) and of mild degeneration (MS≤4). Specimens 6 and 7, however, only contained regions of advanced degeneration. Positivity of chondrons and interterritorial matrix was assessed microscopically for MS≥9 and MS≤4 regions separately. The percentages given at the bottom of the table refer to the fractions of specimens presenting stained chondrons or interterritorial matrix among all analyzed OA cartilage specimens (n=9 in case of MS≥9; n=7 in case of MS≤4). − no staining; + positive; ++ intense staining; / not available; MS: Mankin score

	**PHA-E**				**PHA-L**				**MAA-I**				**SNA**				**ConA**				**PSA**			
Donors	Chondrons		Matrix		Chondrons		Matrix		Chondrons		Matrix		Chondrons		Matrix		Chondrons		Matrix		Chondrons		Matrix	
	MS≤4	MS≥9	MS≤4	MS≥9	MS≤4	MS≥9	MS≤4	MS≥9	MS≤4	MS≥9	MS≤4	MS≥9	MS≤4	MS≥9	MS≤4	MS≥9	MS≤4	MS≥9	MS≤4	MS≥9	MS≤4	MS≥9	MS≤4	MS≥9
1	-	+	-	-	-	-	-	-	-	+	-	+	-	+	-	-	+	+	+	++	+	+	+	++
2	-	-	-	-	-	-	-	-	-	-	-	-	-	-	-	-	+	+	+	++	+	+	+	++
3	-	-	-	-	-	-	-	-	-	-	-	-	-	-	-	-	+	+	+	+	-	-	-	-
4	+	+	-	+	-	+	-	+	-	+	-	+	+	+	+	++	+	+	++	++	-	+	-	++
5	+	+	-	+	-	-	-	-	-	-	-	-	-	+	-	+	+	+	++	++	+	+	++	++
6	/	-	/	+	/	-	/	-	/	-	/	-	/	-	/	-	/	+	/	++	/	+	/	+
7	/	+	/	+	/	+	/	-	/	+	/	+	/	+	/	-	/	+	/	++	/	+	/	+
8	-	+	-	-	-	-	-	-	-	+	-	+	-	-	-	-	+	+	+	++	+	+	+	++
9	-	-	-	-	-	-	-	-	-	-	-	+	-	-	-	-	+	+	+	+	-	-	-	-
positivity (%)	***29%***	***56%***	***0%***	***44%***	***0%***	***22%***	***0%***	***11%***	***0%***	***44%***	***0%***	***56%***	***14%***	***44%***	***14%***	***22%***	***100%***	***100%***	***100%***	***100%***	***57%***	***78%***	***57%***	***78%***

	**DBA**				**PNA**				**JAC**				**LEA**				**VAA**				**ASF**			
Donors	Chondrons		Matrix		Chondrons		Matrix		Chondrons		Matrix		Chondrons		Matrix		Chondrons		Matrix		Chondrons		Matrix	
	MS≤4	MS≥9	MS≤4	MS≥9	MS≤4	MS≥9	MS≤4	MS≥9	MS≤4	MS≥9	MS≤4	MS≥9	MS≤4	MS≥9	MS≤4	MS≥9	MS≤4	MS≥9	MS≤4	MS≥9	MS≤4	MS≥9	MS≤4	MS≥9
1	-	-	-	-	+	+	+	++	-	-	-	-	-	-	-	-	-	-	-	-	+	+	-	+
2	-	-	-	-	+	-	-	+	-	-	-	-	-	-	-	-	-	-	-	-	-	-	-	-
3	-	-	-	-	+	+	-	+	-	-	-	-	-	-	-	-	-	-	-	-	+	+	+	+
4	-	+	+	+	+	+	++	++	-	-	-	++	-	+	-	+	-	+	-	+	-	+	-	++
5	-	-	+	+	+	+	-	+	-	-	-	-	-	-	-	-	-	-	-	-	-	+	+	+
6	/	-	/	-	/	+	/	++	/	-	/	-	/	-	/	-	/	-	/	-	/	+	/	++
7	/	+	/	+	/	+	/	++	/	-	/	+	/	-	/	+	/	+	/	+	/	+	/	++
8	-	-	-	-	+	-	+	++	-	-	-	+	-	+	-	-	-	+	-	+	+	+	-	+
9	-	-	-	-	+	-	-	++	-	-	-	-	-	-	-	-	-	-	-	-	-	+	+	+
positivity (%)	***0%***	***22%***	***29%***	***33%***	***100%***	***67%***	***43%***	***100%***	***0%***	***0%***	***0%***	***33%***	***0%***	***22%***	***0%***	***22%***	***0%***	***33%***	***0%***	***33%***	***43%***	***89%***	***43%***	***89%***
